# SARS-CoV-2 and Arthropods: A Review

**DOI:** 10.3390/v14050985

**Published:** 2022-05-07

**Authors:** Stephen Higgs, Yan-Jang S. Huang, Susan M. Hettenbach, Dana L. Vanlandingham

**Affiliations:** 1 Department of Diagnostic Medicine and Pathobiology, College of Veterinary Medicine, Kansas State University, Manhattan, KS 66506, USA; shiggs@bri.ksu.edu (S.H.); yshuang1985@bri.ksu.edu (Y.-J.S.H.); smhett@bri.ksu.edu (S.M.H.); 2 Biosecurity Research Institute, Kansas State University, Manhattan, KS 66506, USA

**Keywords:** SARS-CoV-2, COVID-19, arthropods, mosquitoes

## Abstract

The emergence of severe acute respiratory syndrome coronavirus-2 (SARS-CoV-2) that led to the unprecedented COVID-19 pandemic exemplifies how a lack of understanding and preparedness for emerging viruses can result in consequences on a global scale. Statements that SARS-CoV-2 could not be transmitted by arthropod vectors were made without experimental support. Here we review laboratory-based research, field studies, and environmental studies to evaluate the potential for the virus to be transmitted either biologically or mechanically by arthropods. Based on these data, we conclude that transmission by arthropods is highly unlikely to play a significant epidemiological role in the transmission of SARS-CoV-2.

## 1. Introduction

The emergence of severe acute respiratory syndrome coronavirus-2 (SARS-CoV-2) that led to the unprecedented COVID-19 pandemic exemplifies how a lack of understanding and preparedness for emerging viruses can result in consequences on a global scale. Coronaviruses are in the subfamily Orthocoronavirinae in the family Coronaviridae in the order Nidovirales [1]. These enveloped viruses have a positive-sense single stranded RNA genome that ranges in size from 26 to 32 kb, one of the largest for an RNA virus [2]. The virus SARS-CoV-2 is related to two other coronaviruses that emerged from animal reservoirs on a much smaller scale: severe acute respiratory syndrome coronavirus (SARS-CoV) and Middle East respiratory syndrome coronavirus (MERS-CoV) [1]. The epidemic of severe respiratory syndrome caused by SARS-CoV began in China during 2002 and subsequently was detected in 17 countries, causing over 8000 cases and over 800 deaths [3,4,5]. Remarkably, the epidemic ended as quickly as it began and no cases have occurred since 2004. It is believed that the viruses emerged from horseshoe bats (e.g., *Rhinolophus sinicus*) with civets as a bridge host to humans [6]. In 2012 another new virus emerged causing Middle East respiratory syndrome but unlike SARS-CoV, the virus responsible for this disease, MERS-CoV, has continued to cause human cases, with a case fatality rate of approximately 35% [7]. Cases have been reported from 27 different countries, most in the Arabian Peninsula, the largest outbreak outside of this region being in the Republic of Korea in 2015. The source of the original human infections was likely camels during close contact with these domesticated animals. But although human infections from camels may continue to occur, many human infections likely result due to person-to-person transmission with over 2500 laboratory-confirmed cases to date [8]. Due to the relationship between SARS-CoV-2, SARS-CoV and MERS-CoV, it was therefore reasonable to assume that SARS-CoV-2 would share common characteristics with these other betacoronaviruses, including the route(s) of transmission and the potential involvement of animal reservoirs. Indeed, as with the viruses that cause SARS and MERS, SARS-CoV-2 is spread through aerosol and close contact with infected people and can be transmitted to other susceptible animals.

## 2. Discussion

Such extrapolation may be logical, however; the emergence of new viruses and introduction of known viruses into areas outside of their normal endemic range can provide new opportunities and challenges that can result in deviations from expected epidemiological patterns and evolution of new virus variants with novel phenotypes including hitherto unseen pathologies. As observed with the introduction of West Nile virus (WNV) into the United States in 1999, the dispersal of chikungunya virus (CHIKV) through the archipelagoes along the Indian Ocean Islands in 2005, and the emergence of Zika virus (ZIKV) in 2015, even well-studied zoonotic viruses have the ability to infect species previously not known to be involved in documented transmission cycles and to utilize new modes of transmission. In the United States, WNV established its transmission cycles, being detected in over 60 species of mosquitoes and with multiple vertebrate hosts that it had previously not been in contact with. The spread of CHIKV from East Africa to islands in the Indian Ocean was in part due to a E2-A226V mutation that made it more infectious to the widely distributed Asian tiger mosquito *Aedes albopictus* [9]. Within a few years, CHIKV spread to, and become endemic throughout Southeast Asia and the Americas. As ZIKV spread from Africa, the involvement of large human populations revealed hitherto unseen pathologies including fetal microcephaly.

In the early phase of the COVID-19 pandemic, the World Health Organization, and news and social media outlets, stated that the transmission of SARS-CoV-2 by mosquitoes or any other arthropod species was not possible or highly unlikely [10,11,12,13]. These unsubstantiated statements were made due to the fact that historically coronaviruses rarely if ever have been isolated from arthropod vectors. Runde virus, which was isolated from ticks associated with seabirds [14], was described as “coronavirus-like” due to its morphological characteristics, but “some characteristics of the virus differ considerably from those of known coronaviruses”. To date, the taxonomic classification of Runde virus remains unclear and since it is not listed as a coronavirus by the International Committee on Taxonomy of Viruses, one could say that no known coronavirus is infectious to and transmitted by arthropods. For viruses to infect and ultimately be transmitted by hematophagous arthropods such as mosquitoes, the arthropods must typically feed on a vertebrate host that is viremic. Susceptibility to infection may be arthropod species specific but also may require that a threshold level of viremia be met or exceeded [15]. Although SARS-CoV-2 RNA has been detected in plasma, the lack of transmission through transfusion of human blood products would suggest that infectious virus is absent or present at low levels in blood [16]. In support of this, studies in non-human primates did not detect infectious SARS-CoV-2 in blood [17]. Based on the cited studies that suggest that SARS-CoV-2 infected people have little if any sustained viremia, the statements that transmission by mosquitoes was not possible or highly unlikely may have made sense. Nonetheless, when the statements were made, critical experimental vector competence studies with SARS-CoV-2 had not been performed. Another reason for assuming that hematophagous arthropods would not become infected with SARS-CoV-2 is that the virus mainly replicates in tissues of the respiratory tract [18]. Due to a lack of knowledge related to SARS-CoV-2 environmental stability and presence in body fluids and fomites [19], the potential for virus dissemination and mechanical transmission by arthropods was unknown. In the one Chinese study [20] that tested wild-caught *Culex* and *Anopheles* mosquitoes, no SARS-CoV-2 virus was detected. As described below and in Table 1, several studies have now been performed to qualify those early statements that SARS-CoV-2 is not transmitted by arthropods.

### 2.1. Biological Transmission

The first published vector competent study found no evidence that SARS-CoV-2 was able to replicate in three species of mosquitoes, *Aedes aegypti*, *Ae. albopictus*, and *Culex quinquefasciatus*, that receive relatively high quantities of infectious virus via intrathoracic injection [21]. This extreme approach to challenge mosquitoes was used since it is known that by circumventing the normal infection process of hematophagous arthropods can sometimes enable a virus to infect an incompetent mosquito species or even non-hematophagous insects [22,23,24,25]. The assumption was that if this approach failed to infect the mosquitoes, then oral exposure to virus in blood would most certainly not result in infection. As described below, this assumption has proven to be valid. Since following intrathoracic inoculation, SARS-CoV-2 failed to persist or replicate in these mosquitoes, the potential for biological transmission of SARS-CoV-2 by these mosquitoes was effectively negated.

A subsequent publication [26] demonstrated that *Cx. quinquefasciatus*, *Cx. tarsalis*, and the midge *Culicoides sonorensis* did not become infected after feeding on blood contaminated with SARS-CoV-2. This study also demonstrated that insect-derived cell lines were not permissive to SARS-CoV-2 replication. A European study used *Ae. albopictus* and *Cx. pipiens* [27] that were presented with infected blood meals. Mosquitoes were collected at 0-, 3-, 7-, and 10-days post-feeding and then bodies and legs and wings were tested by RT-qPCR to determine infection and dissemination rates respectively. Based on the data, the authors reported that these mosquito species could not be infected when orally challenged with SARS-CoV-2 in a blood meal. Interestingly, mosquitoes that fed on the infected meals were allowed to lay eggs, that were subsequently hatched to produce larva, some of which were maintained to produce adults. Larvae and adults tested by RT-qPCR for virus were all negative, and it was concluded that under these experimental conditions, SARS-CoV-2 was not vertically transmitted.

When cell lines derived from mosquitoes (C6/36 from *Ae. albopictus*, Aag2 from *Ae. aegypti*, HSU from *Cx. quinquefasciatus*, TrR2 from *Cx. tarsalis*, W8a from *Culicoides sonorensis*, Sf9 from *Spodoptera frugiperda*) were exposed to SARS-CoV-2, none became infected with the virus [20,26]. These negative results may be extrapolated with caution to further suggest that arthropods are not susceptible to infection with SARS-CoV-2. To date, cells derived from *Drosophila melanogasther*, in which an angiotensin-converting enzyme (ACE) has been described, has not been tested for SARS-CoV-2 susceptibility, even though ACE2 is a known receptor for the virus [20].

Several species of domesticated and wild vertebrates, including dogs and cats [28,29] and white-tailed deer [30,31] are susceptible to infection with SARS-CoV-2. Villar et al. [32] posed the following question: could ectoparasites such as fleas and ticks be involved in the transmission of SARS-CoV-2? No challenge experiments were performed, but coronavirus sequences were detected in laboratory reared and unfed cat fleas, *Ctenocephalus felis*, collected from two feral cats. How these sequences came to be present was unexplained.

### 2.2. Mechanical Transmission

SARS-CoV-2 possesses several common characteristics with pathogenic viruses that can be spread through mechanical transmission including the presence of infectious virions in body fluids secreted by infected hosts [33] and relatively high environmental stability [34,35,36]. Several studies have demonstrated that SARS-CoV-2 may remain infectious for several hours or longer, depending upon temperature, relative humidity, and characteristics of the surface [37]. Subsequently, several arthropod species have been examined for the ability to support the mechanical transmission of SARS-CoV-2. The experiments also investigated the potential for mechanical transmission by *Ae. albopictus* by allowing them to probe several times on an infected blood meal (1.2 × 10^6^ PFU/mL) and then immediately allowing them to feed on uninfected blood that was analyzed for the virus. The virus was not detected in the second bloodmeal and it was concluded that the species was “unable to mechanically transmit the virus to a healthy host after first feeding on a SARS-CoV-2-positive host, even in the hypothetical case of very high viremia”. Virus was detected in *Ae. albopictus* but the low viral load (6.32 × 10^2^ to 3.44 × 10^1^ plaque forming unit equivalents) limits the possibility of mechanical transmission [27]. Balaraman et al. [38] exposed house flies, *Musca domestica*, to milk contaminated with SARS-CoV-2 and demonstrated that flies could remain positive for up to 24 h post-exposure and that viral RNA could be detected on surfaces with which they had come into contact. However, infectious virus could not be detected on these surfaces, and they concluded that the low level of infectious virus carried by flies limits their capability for SARS-CoV-2 transmission. Since SARS-CoV-2 has been detected in human feces [39,40], Montes et al. [41] posed the question as to whether or not houseflies might mechanically transmit SARS-CoV-2 from contaminated feces but performed no experiments to address this question.

In a recent study, over 1345 arthropods were collected from the houses of SARS-CoV-2-infected people [42]. Insects from 11 Dipteran genera (Asilidae, Calliphoridae, Ulidiidae, Dolichopodidae, Drosphilidae, Muscidae, Phoridae, Psychodidae, Sarcophidae, Syrphidae, Tabanidae) and cockroaches (*Blatta* spp.) were assigned to 243 pools and tested by PCR but none were positive [43]. Interestingly, from some of these houses, SARS-CoV-2 was detected in the pets of these owners [28].

## 3. Conclusions

Based on experimental data and observations from nature, SARS-CoV-2 is unable to infect and be biologically transmitted by hematophagous arthropods. Since it remains unknown how wild animals are becoming infected, further studies on ectoparasites may, however, be warranted. Although the virus may remain infectious on some surfaces, and be present in human secretions and feces, there are similarly no data to suggest that mechanical transmission of the virus by arthropods play a significant, if any role, in virus transmission and human infection.

## Figures and Tables

**Table 1:** Summary of published studies examining the role of arthropod species in the transmission of SARS-CoV-2.

Species	Techniques and Major Findings	References
Mosquito: *Ae. aegypti*, *Ae. albopictus* and *Cx. quinquefasciatus*	Intrathoracic injection Biological transmission is highly unlikely	[21]
Mosquito: *Ae. albopictus* and *Cx. pipiens*	Blood feeding Biological and mechanical transmissions are both highly unlikely	[21,27]
Mosquito: *Anopheles* spp. and *Culex* spp.	Field caught mosquitoes with no positive detection	[20]
Mosquito: *Culex tarsalis*	Blood feeding Biological and mechanical transmissions are both highly unlikely	[26]
Midge: *Culicoides sonorensis*	Blood feeding Biological and mechanical transmissions are both highly unlikely	[26]
Housefly: *Musca domestica*	Direct contact with contaminated materials Mechanical transmission is highly unlikely	[38]
*Musca domestica*, *Cochliomyia macellaria*, *Phormia regina* and unidentified species of Diptera in the genera: Asilidae, Calliphoridae (*including Lucilia* spp., *Cochliomyia* spp.), Ulidiidae, Dolichopodidae, Drosphilidae, Muscidae, Phoridae, Psychodidae, Sarcophidae, Syrphidae, and Tabanidae.	Field caught arthropods with no positive detection	[43]
Cockroach: *Blatta* spp.	Field caught arthropods with no positive detection	[43]
Flea: *Ctenocephalus felis*	Detection of coronavirus-like sequences Biological implications unknown	[32]

## Data Availability

Not applicable.
